# MALDI imaging reveals NCOA7 as a potential biomarker in oral squamous cell carcinoma arising from oral submucous fibrosis

**DOI:** 10.18632/oncotarget.11046

**Published:** 2016-08-04

**Authors:** Xiaoyan Xie, Yuchen Jiang, Yao Yuan, Peiqi Wang, Xinyi Li, Fangman Chen, Chongkui Sun, Hang Zhao, Xin Zeng, Lu Jiang, Yu Zhou, Hongxia Dan, Mingye Feng, Rui Liu, Qianming Chen

**Affiliations:** ^1^ State Key Laboratory of Oral Diseases, West China Hospital of Stomatology, Sichuan University, Chengdu 610041, China

**Keywords:** NCOA7, MALDI Imaging, oral squamous cell carcinoma, oral submucous fibrosis, aryl hydrocarbon receptor

## Abstract

Oral squamous cell carcinoma (OSCC) ranks among the most common cancer worldwide, and is associated with severe morbidity and high mortality. Oral submucous fibrosis (OSF), characterized by fibrosis of the mucosa of the upper digestive tract, is a pre-malignant lesion, but the molecular mechanisms underlying this malignant transformation remains to be elucidated. In this study, matrix-assisted laser desorption ionization imaging mass spectrometry (MALDI-IMS)-based proteomic strategy was employed to profile the differentially expressed peptides/proteins between OSCC tissues and the corresponding adjacent non-cancerous OSF tissues. Sixty-five unique peptide peaks and nine proteins were identified with altered expression levels. Of them, expression of NCOA7 was found to be up-regulated in OSCC tissues by immunohistochemistry staining and western blotting, and correlated with a pan of clinicopathologic parameters, including lesion site, tumor differentiation status and lymph node metastasis. Further, we show that overexpression of NCOA7 promotes OSCC cell proliferation in either *in vitro* or *in vivo* models. Mechanistic study demonstrates that NCOA7 induces OSCC cell proliferation probably by activating aryl hydrocarbon receptor (AHR). The present study suggests that NCOA7 is a potential biomarker for early diagnosis of OSF malignant transformation, and leads to a better understanding of the molecular mechanisms responsible for OSCC development.

## INTRODUCTION

Oral squamous cell carcinoma (OSCC) is among the most common cancer worldwide, and is associated with severe morbidity and high mortality. OSCC patients are usually detected when cancers have proceeded to an advanced stage, which results in poor prognosis and low survival rate of this disease [1]. Despite therapeutic improvement, the five-year survival rate of patients with OSCC remains below 50% [2]. Most of OSCC tumors arise from clinically evident oral potentially malignant disorders (OPMDs), such as oral submucous fibrosis (OSF), oral lichen planus (OLP) and oral leukoplakia (OLK), and the malignant transformation of these OPMDs rate from 1.4% to 36% [3–5].

OSF is a pre-cancerous condition characterized by chronic inflammation, epithelial atrophy as well as loss of rete ridges due to excessive deposition of collagen [6]. It has been well accepted that OSF is a persistent public health threat for many parts of the world, including Southeast Asia, South Africa and the UK, due to the habit of areca nut chewing. Although 2.3%-12% of OSF may finally transform into invasive OSCC [7], a reliable diagnostic strategy monitoring early malignant transformation of OSF is still lacking. Therefore, a better understanding of the key molecular events during this pathological process, including identifying proteins with abnormal expression or modification patterns, is still needed.

The proteome is the entire set of proteins expressed by a whole genome, however, proteome is more complex and dynamic compared to the genome. Proteomic analyses, which aim to determine the expression, modification or interaction of proteins, become powerful tools to decipher the mechanisms underlying the development of diverse human diseases [8]. Matrix-assisted laser desorption ionization imaging mass spectrometry (MALDI-IMS) has emerged in the recent years as a tissue-based approach that can address the inherent protein expression heterogeneity of tissues by maintaining tissue integrity, histopathology features, and analyte distribution across the tissue [9]. In comparison to other proteomics approaches, such as liquid chromatography-tandem mass spectrometry (LC-MS/MS) or two-dimensional alpolyacrylamide gel electrophoresis (2D-E), MALDI-IMS is more powerful in testing clinical tissue samples, since it can be used in combination with microscopy, enabling spatially resolved, label-free imaging of various peptides/proteins in their histological context and visualizing the distribution of hundreds of molecular compounds in a frozen unprocessed tissue section by a single measurement [10, 11]. Clusters of proteins revealed by MALDI-IMS have been demonstrated as prognostic and/or diagnostic indicators in several tumors, including OSCC [12–15]. In this report, by MALDI-IMS based proteomic strategy, we compare proteins expression profile between OSCC tissues versus adjacent non-cancerous OSF tissues. Sixty-five peaks are identified with changed expression level, and among them, nine proteins are positively identified. Bioinformatics shows that NCOA7 is associated with several known key onco-proteins in OSCC development. Further, immunostaining and western blotting demonstrate that NCOA7 is markedly up-regulated in OSCC tissues, compared to OSF tissues, and NCOA7 expression is positively related to the lesions site, histological degree and lymph node metastasis, respectively. Moreover, we show that NCOA7 overexpression promotes OSCC cell proliferation, which is probably by activating the transcriptional capability of aryl hydrocarbon receptor (AHR).

## RESULTS

### MALDI imaging

**Figure 1:**
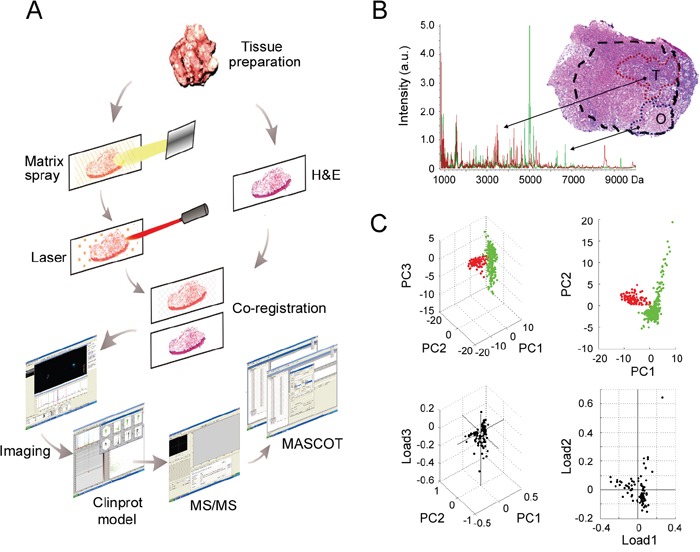
MALDI-IMS analyses of OSCC and adjacent non-cancerous OSF areas **A.** Schematic illustrating of the workflow of MALDI-IMS analyses of clinical tissues. **B.** An overlapped MALDI spectral profiles obtained from OSCC areas (red peaks) and OSF areas (green peaks), and representative H&E staining imaging for OSCC areas (T) and the adjacent OSF areas (O). T, tumor; O, OSF; a.u., arbitrary unit. **C.** PCA analyses were performed to evaluate the multidimensional distributions of the identified peaks. Peaks detected from OSCC or OSF areas were labeled as red or green dots, respectively

**Figure 2:**
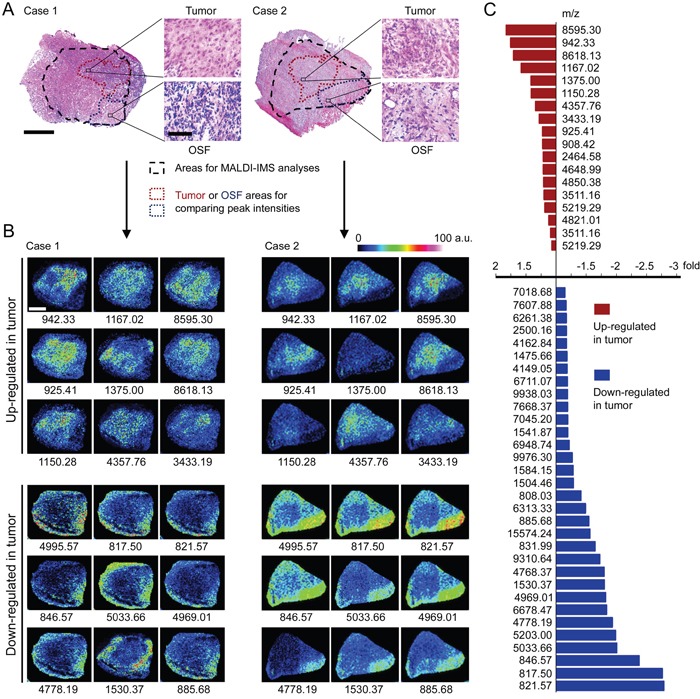
Altered peptide peaks identified by MALDI-IMS **A.** Representative H&E staining imaging for OSCC areas and the adjacent OSF areas. The areas selected for MALDI-IMS analyses was indicated by black dotted line. The OSCC and OSF areas used for calculating altered peaks were indicated by dark red or blue dotted line, respectively. Scale bar: original images, 250 μm; enlarged images, 50 μm. **B.** Representative MALDI-MS images of 18 peaks with most significant alterations. Sample Case 1 and Case 2 are the same samples as shown in (A), respectively. The m/z value of each peak was labeled under the corresponding image. a.u., arbitrary unit. **C.** Changed intensities of 50 identified peaks.

The peak profiles from ten clinical samples were obtained by MALDI-IMS and the principle workflow was summarized in Figure 1A. Representative MS spectrums from OSCC (red) and adjacent non-cancerous OSF (green) areas were shown in Figure 1B. Principal component analysis (PCA) was performed on the raw spectral data and pooled together a maximum amount of variance in a minimum number of independent variables to understand the histological heterogeneity of the identified peaks. As shown in Figure 1C, the peaks in OSCC areas statistically differed from those peaks in adjacent non-cancerous OSF areas. Further, the intensity of each peak was normalized according to the average ionic intensities, and then compared by ClinprotTools 3.0 software. As shown in Table 1, 23 peaks were higher in OSCC areas (*P* < 0.05), whereas 42 peaks were higher in OSF areas (*P* < 0.05). A great proportion of these changed peaks were within m/z range 800-1500 and 4200-5000. The representative H&E staining images and MALDI-IMS images of 18 peaks with most significant alterations (9 up-regulated and 9 down-regulated in OSCC areas) were shown in Figure 2A–2B, respectively. In addition, the altered levels of fifty peaks were shown in Figure 2C.

**Table 1:** Altered peaks identified by MALDI-IMS

Peak No.	m/z	Alteration ^a^	Mean Intensity in OSCC (a.u.) ^b^	Mean Intensity in OSF (a.u.)
1	8595.30	↑	2.00	1.09
2	942.33	↑	5.46	3.10
3	8618.13	↑	1.85	1.08
4	1167.02	↑	2.52	1.59
5	1375.00	↑	1.88	1.32
6	1150.28	↑	2.37	1.67
7	4357.76	↑	2.32	1.72
8	3433.19	↑	1.79	1.39
9	925.41	↑	4.22	3.42
10	908.42	↑	2.39	1.94
11	2464.58	↑	2.04	1.67
12	4648.99	↑	1.71	1.40
13	4850.38	↑	1.58	1.30
14	3511.16	↑	1.97	1.63
15	5219.29	↑	1.47	1.23
16	4821.01	↑	1.69	1.50
17	3394.69	↑	1.92	1.75
18	5092.91	↑	1.42	1.32
19	5364.13	↑	1.33	1.24
20	3816.48	↑	1.58	1.50
21	5723.70	↑	1.22	1.16
22	6036.99	↑	1.32	1.27
23	3411.89	↑	1.68	1.64
24	6283.52	↓	1.11	1.17
25	5052.98	↓	1.59	1.69
26	6212.12	↓	1.09	1.16
27	6395.28	↓	1.05	1.13
28	3466.34	↓	1.75	1.89
29	8503.07	↓	0.96	1.06
30	6470.70	↓	1.06	1.18
31	6991.26	↓	0.96	1.07
32	7018.68	↓	0.99	1.12
33	7607.88	↓	0.92	1.06
34	6261.38	↓	1.22	1.43
35	2500.16	↓	1.70	2.00
36	4162.84	↓	1.52	1.79
37	1475.66	↓	1.57	1.85
38	4149.05	↓	1.55	1.84
39	6711.07	↓	1.01	1.20
40	9938.03	↓	0.83	0.99
41	9907.42	↓	0.82	0.98
42	7668.37	↓	0.91	1.09
43	7045.20	↓	1.00	1.20
44	1541.87	↓	1.50	1.80
45	6948.74	↓	0.99	1.21
46	9976.30	↓	0.84	1.07
47	1584.15	↓	1.74	2.24
48	1504.46	↓	1.64	2.12
49	808.03	↓	1.70	2.41
50	6313.33	↓	1.11	1.66
51	885.68	↓	2.00	3.10
52	15574.24	↓	1.81	2.84
53	831.99	↓	1.56	2.58
54	9310.64	↓	0.78	1.35
55	4768.37	↓	1.42	2.56
56	1530.37	↓	1.65	2.98
57	4969.01	↓	1.91	3.49
58	6687.47	↓	1.02	1.88
59	4778.19	↓	1.47	2.85
60	5203.00	↓	1.34	2.67
61	5033.66	↓	1.76	3.54
62	846.57	↓	1.51	3.60
63	817.50	↓	1.57	4.35
64	821.57	↓	1.46	4.08
65	4995.57	↓	2.62	31.52

a ↑, up-regulated in OSCC; ↓, Down-regulated in OSCC.

b arbitrary unit

### Protein identification

Among the 65 identified peaks, 20 peaks with high intensity and signal/noise (S/N) value were manually selected and subjected to MS/MS identification. The raw MS/MS data was processed by searching against Mascot service and/or ExPASy protein sequence database. As results, 9 distinct proteins were positively identified, including MAAI (m/z 817.50), CLCA2 (821.57), DJC14 (831.99), NCK5L (846.57), CAPSD (885.68), NCOA7 (942.33), RUVB2 (1167.02), CLCN6 (1530.37) and TEX2 (1557.24). The detailed information of these identified proteins, including peptide sequence, Uniprot access No. and protein description, were listed in Table 2.

**Table 2:** Proteins identified in MS/MS

Peak No.	m/z	Peptide Sequence^a^	Uniprot No.	Protein description	Gene name	Mw^b^	pI^c^	Alteration In OSCC^d^
1	817.50	ALNPMKQ	O43708	Maleylacetoacetate isomerase	MAAI	24,212	8.8	↓
2	821.57	SNSAVPPA	Q9UQC9	Calcium-activated chloride channel regulator 2	CLCA2	103,941	6.56	↓
3	831.99	SSNFCH	Q6Y2×3	DnaJ homolog subfamily C member 14	DJC14	78,569	8.37	↓
4	846.57	GPGKSGESAG	Q9HCH0	Nck-associated protein 5-like	NCK5L	139,013	8.45	↓
5	885.68	NPAIDDGKG	A4GZ97	Capsid protein	CAPSD	80,268	9.79	↓
6	942.33	LYNDISH	Q8NI08	Nuclear receptor coactivator 7	NCOA7	106,162	5.42	↑
7	1167.02	LGTKMIESL	Q9Y230	RuvB-like 2	RUVB2	51,157	5.49	↑
8	1530.37	PYMNPSPFTVSPN	P51797	Chloride transport protein 6	CLCN6	97,289	6.39	↓
9	1557.24	KTSSSSPLSSP	Q8IWB9	Testis-expressed sequence 2 protein	TEX2	125,303	5.71	↓

a The peptide sequence was calculated by searching the raw MS/MS data with MASCOT online service.

b Theoretical Mw was calculated by the EXPASY Compute pI/Mw tool.

c Theoretical pI was calculated by the EXPASY Compute pI/Mw tool.

d ↑, up-regulated in OSCC; ↓, down-regulated in OSCC.

### Bioinformatics analysis

**Figure 3:**
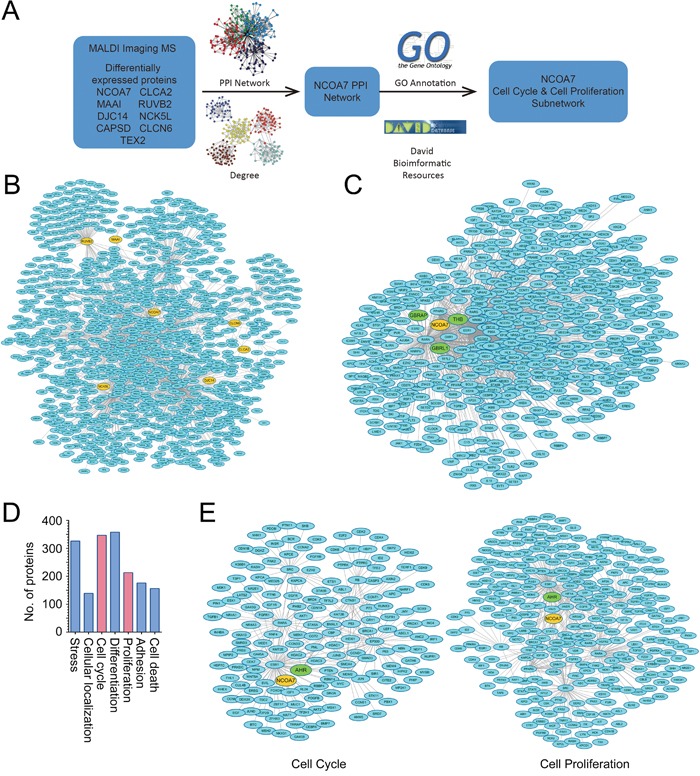
Bioinformatics analysis of proteins identified from MALDI-IMS **A.** Schematic illustrating of the workflow of bioinformatics analyses. **B.** Total PPI network was established for the nine proteins identified from MALDI-IMS. **C.** Sub-PPI network for NCOA7-asscoiated proteins was extracted from the total network **D.** Predicted NCOA7-associated proteins were divided into several groups based on their function. **E.** Predicted NCOA7-associated proteins involved in the regulation of cell cycle (left panel) and cell proliferation (right panel).

To explore the potential roles of the changed proteins in OSCC development, PPI network and functional annotation was further performed (Figure 3A). PrePPI, which was established based on the total 1109 predicted proteins related with the nine proteins identified from MALDI-IMS, was used to construct the PPI network. As results, a total number of 1839 paired PPIs were addressed (Figure 3B). The NCOA7-associated proteins were then extracted from the total network (Figure 3C), and was further modified by GO annotation functional cluster analysis (Figure 3D). Interestingly, a significant proportion of NCOA7-associated proteins were involved in the regulation of cell cycle and proliferation (Figure 3E). The cell cycle subnetwork included 254 paired PPIs addressing 166 unique proteins (Figure 3E left panel), while the cell proliferation subnetwork included 442 paired PPIs addressing 284 proteins (Figure 3E right panel). Considering that the accelerated cell cycle progression and unlimited cell proliferation are hallmarks of cancer cells, NCOA7 was selected for further studies.

### NCOA7 expression is correlated with OSCC development

**Figure 4:**
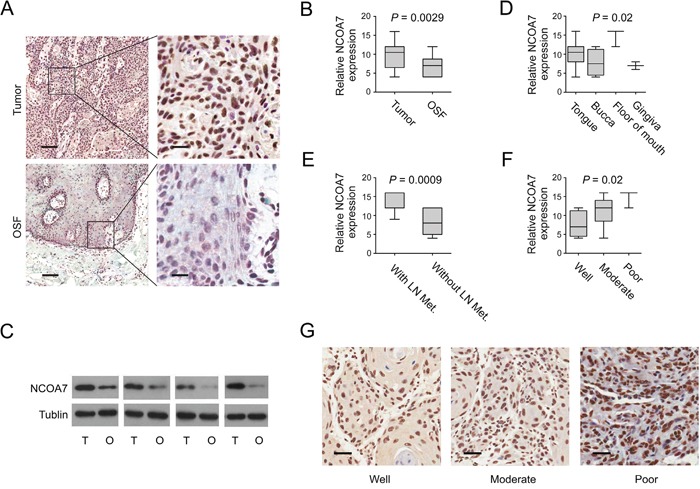
NCOA7 up-regulation is correlated with OSCC development **A.** Representative images of NCOA7 immunostaining of OSCC tissues and OSF tissues. Scale bar: left panels, 500 μm; right panels, 100 μm. **B.** NCOA7 immunostaining scores in OSCC tissues and OSF tissues were analyzed. **C.** NCOA7 expression between OSCC and OSF samples was examined by western blot. T: tumor, O: OSF **D.** NCOA7 immunostaining scores in different tumor lesion sites were analyzed. **E.** NCOA7 immunostaining scores in tumors with or without lymph node metastasis were analyzed. LN Met, lymph node metastasis. **F.** NCOA7 immunostaining scores in well, moderately or poorly differentiated tumors were analyzed **G.** Representative images of NCOA7 immunostaining in well, moderately or poorly differentiated tumors. Scale bar: 100 μm.

To verify whether our findings by MALDI-IMS and bioinformatics were of clinical relevance, expression of NCOA7 between OSCC and OSF tissues was examined. Clinicopathologic information for the clinical samples was summarized in Table 3. As shown in Figure 4A, NCOA7 immunoreactivity was mainly detected in the nucleus. Notably, expression of NCOA7 was markedly elevated in OSCC tissue compared to OSF counterparts (*P* = 0.0029, Figure 4A–4B). Similarly, upregulation of NCOA7 was also observed by western blots (Figure 4C).

**Table 3:** Correlation between NCOA7 expression and OSCC clinicopathologic parameters

Group	Number	NCOA7		*P* value
		Mean	Standard deviation	
Age			0.46	
≤ 40	6	10.17	4.12	
40 – 60	10	9.09	4.48	
≥ 60	4	11.50	4.12	
Lesion site				0.02^*^
Tongue	11	11.40	2.95	
Bucca	4	7.75	3.50	
Floor of mouth	3	14.67	2.31	
Gingiva	2	7.00	1.41	
Histological degree				0.02^*^
Well differentiation	8	7.63	3.20	
Moderate Differentiation	9	10.89	3.86	
Poor differentiation	3	14.67	2.31	
Tumor size (cm)				0.59
≤ 2	2	10.50	2.12	
2 – 4	7	11.14	4.26	
≥ 4	11	9.09	3.94	
Lymph node metastasis				
Yes	7	13.86	2.85	0.0009^*^
No	13	8.15	3.18	

* *P* < 0.05 statistically significant

We also examined correlation between NCOA7 expression and a series of clinicopathologic parameters. Interestingly, NCOA7 immunoreactivity was more intense in those tumor tissues from tongue or floor of mouth, compared to bucca or gingiva (*P* = 0.02, Table 3; Figure 4D). Further, a high level of NCOA7 expression was more likely to be associated with lymph node metastasis (*P* < 0.0001, Table 3; Figure 4E). In contrast to well differentiated tumor, the level of NCOA7 was higher in the poorly and moderately differentiated (*P* = 0.02, Table 3; Figure 4F–4G). However, no correlation was observed between NCOA7 expression and tumor size or patient age (Table 3).

### Overexpression of NCOA7 induces proliferation in OSCC cells

**Figure 5:**
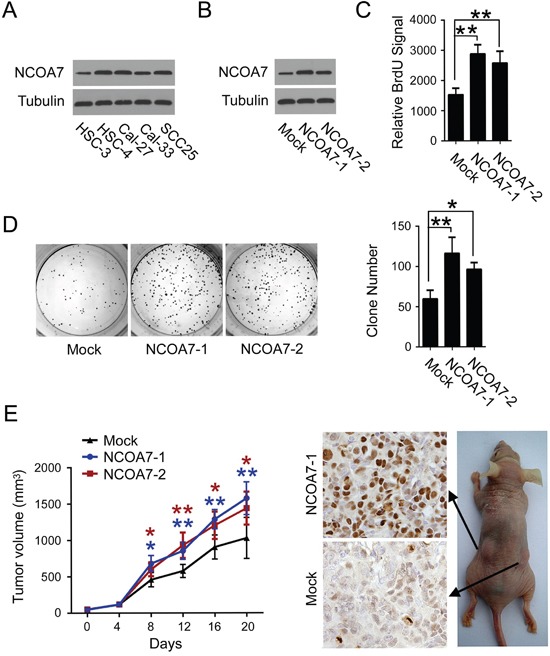
Overexpression of NCOA7 induces proliferation in OSCC cells **A.** Expression of NCOA7 in several OSCC cell lines was examined by western blot. **B.** Expression of NCOA7 in normal HSC-3 cells and two NCOA7-overexpressed sub-clones was examined by western blot. **C.** Proliferation of normal HSC-3 cells and two NCOA7-overexpressed sub-clones was examined by BrdU labeling assay. **D.** Proliferation of normal HSC-3 cells and two NCOA7-overexpressed sub-clones was examined by colony formation assay. **E.** Mean tumor volume (left panel) and Ki67 expression (right panel) in tumors after subcutaneous transplantation of normal HSC-3 cells or two NCOA7-overexpressed sub-clones (n = 8). Blue asterisks, NCOA7-1 group versus mock group; dark red asterisks, NCOA7-2 group versus mock group. All data were representative of at least three independent experiments. ***, *P* < 0.001; **, *P* < 0.01; *, *P* < 0.05.

Since a cluster of NCOA7-associated proteins were involved in regulation of cell cycle and cell proliferation, to examine the potential role of NCOA7 in OSCC development, the effect of NCOA7 expression on OSCC cell proliferation was tested. As a pilot study, NCOA7 expression in a pan of OSCC cell lines was examined. High NCOA7 expression was observed in HSC-4, Cal-27 and SCC25 cell lines, while the level of NCOA7 expression was relatively low in HSC-3 and Cal-33 cell lines (Figure 5A). Therefore, NCOA7 was stably overexpressed in HSC-3 cell line, and two sub-clones, NCOA7-1 and NCOA7-2, were selected (Figure 5B). As results, NCOA7 induced about 2-fold augmentation in BrdU labeling assay (Figure 5C) and 1.8-fold more colonies in the colony formation assay (Figure 5D). Similarly, siRNA-mediated NCOA7 knockdown markedly reduced the proliferative rate of HSC-4 cells (Supplementary Figure S1A-C).

To determine the impact of NCOA7 on tumor growth *in vivo*, HSC-3 cells stably overexpressing NCOA7 or mock vector were subcutaneously injected into nude mice. As shown in Figure 5E, though no difference was observed for the first 8 days, the growth of those tumors arising from both NCOA7-overexpressed sub-clones, was much faster than mock control groups. Accordingly, more Ki-67-positive cells were observed in either NCOA7-1 or NCOA7-2-formed tumors. These results indicated that NCOA7 had a profound effect in mediating OSCC cell proliferation both *in vitro* and *in vivo*.

### NCOA7 regulates cell proliferation by activating aryl hydrocarbon receptor (AHR)

**Figure 6:**
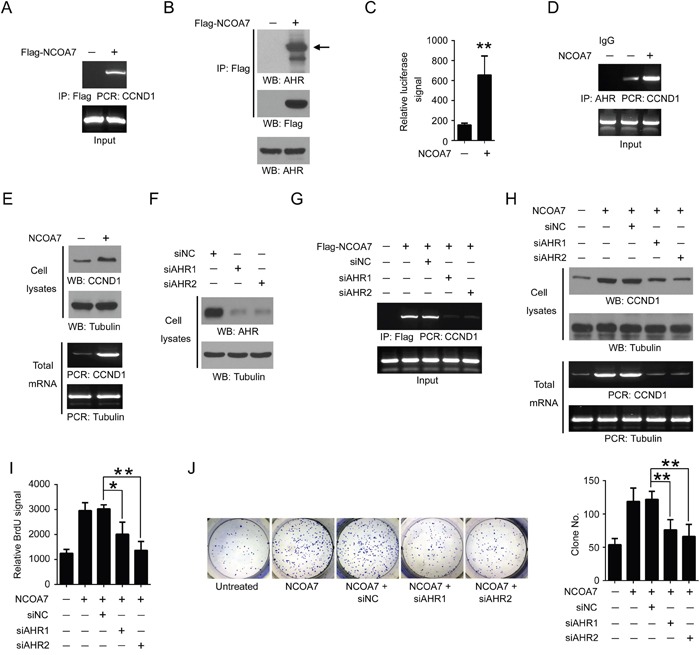
NCOA7 regulates cell proliferation by activating AHR **A.** HSC-3 cells were overexpressed with Flag-NCOA7 or mock vector. NCOA7 binding to CCND1 promoter was examined by ChIP assay. **B.** HSC-3 cells overexpressed with Flag-NCOA7 or mock vector. NCOA7 binding to endogenous AHR was examined using nuclear extracts by Co-IP. **C.** HSC-3 cells were overexpressed with NCOA7 or mock vector. The transcriptional activity of AHR was examined by luciferase assay. **D.** HSC-3 cells were overexpressed with NCOA7 or mock vector. AHR binding to CCND1 promoter was examined by ChIP assay. **E.** HSC-3 cells overexpressed with Flag-NCOA7 or mock vector. Expression of CCND1 was examined by Western blot and RT-PCR. **F.** HSC-3 cells were transfected with siAHR1, siAHR2 or negative control siRNA (siNC), respectively. Expression of AHR was examined by Western blot. **G.** HSC-3 cells were transfected with Flag-NCOA7, siAHR1, siAHR2 and/or siNC. NCOA7 binding to CCND1 promoter was examined by ChIP assay. **H.** HSC-3 cells were transfected with Flag-NCOA7, siAHR1, siAHR2 and/or siNC. Expression of CCND1 was examined by Western blot and RT-PCR. **I.** HSC-3 cells were transfected with Flag-NCOA7, siAHR1, siAHR2 and/or siNC. Cell proliferation was examined by BrdU labeling assay. **J.** HSC-3 cells were transfected with Flag-NCOA7, siAHR1, siAHR2 and/or siNC. Cell proliferation was examined by colony formation assay. All data were representative of at least three independent experiments. ***, *P* < 0.001; **, *P* < 0.01; *, *P* < 0.05.

Next, we sought to explore the mechanisms responsible for NCOA7-mediated OSCC cell proliferation. We noticed that potential interconnection between NCOA7 and AHR was predicted in our aforementioned bioinformatics analyses (Figure 3E). It is reported that AHR functioned in cell cycle regulation and was implicated in hepatoma carcinogenesis [16]. Strikingly, chromatin immunoprecipitation (ChIP) showed that NCOA7 bonded to the promoter region of CCND1, an AHR- target gene (Figure 6A) [17]. Further, endogenous AHR was detected in the Flag-NCOA7 immuno-precipitant from HSC-3 cell nuclear extracts (Figure 6B), suggesting that these two proteins can bind to each other in the nucleus. Therefore, of our particular interest, we examined the impact of NCOA7 on AHR expression and activity. In spite of no changes in AHR expression (data not shown), the transcriptional activity of AHR was apparently elevated after NCOA7 expression by luciferase assay (Figure 6C). In line with this, overexpression of NCOA7 also enhanced AHR binding to CCND1 promoter and indeed increased CCND1 expression at both mRNA and protein levels (Figure 6D–6E). These results suggested that NCOA7 was a potential nuclear co-activator of AHR.

To determine the role of AHR in NCOA7-mediated OSCC cell proliferation, expression of AHR was inhibited by two distinct siRNAs (Figure 6F). As expected, loss of AHR blocked NCOA7 binding to CCND1 promoter (Figure 6G), and largely abolished NCOA7-induced CCND1 expression (Figure 6H). Notably, NCOA7-indcued OSCC cell proliferation was substantially attenuated after AHR knockdown, revealed by both BrdU labeling assay (Figure 6I) and colony formation assay (Figure 6J). These results suggested that NCOA7 promoted OSCC proliferation probably by activating AHR.

## DISCUSSION

Proteomic analyses have been widely applied in cancer researches, since they allow high-throughput screening of a broad variety of molecules from either experimental or clinical samples. In the past decades, studies using traditional proteomic strategies, such as two-dimensional alpolyacrylamide gel electrophoresis (2-DE)-based strategy and tag labeling-based quantitative strategy, have identified a large body of diagnostic biomarkers and therapeutic targets. However, complex sample preparation procedures, including tissue homogenization, protein extraction, electrophoresis and tryptic digestion, are routinely required for these approaches, and thus probably lead to artificial bias in the subsequent mass spectrometry analyses [18]. Therefore, detailed and accurate profiling of tumor-specific proteins and modifications is still challenging. MALDI-IMS is an emerging technology for analyzing spatial distribution of both proteins and small molecules [19]. For MALDI-IMS, tissue samples remain intact, avoiding pre-analytical protein changes resulted from homogenization and extraction [20]. Moreover, selection of interested histologic or pathological areas of tissue samples is based on H&E staining. Thus, it is more reliable than surgical tissue discrimination, which is widely used for traditional proteomic strategies. In this study, MALDI-IMS was utilized to characterize the differentially expressed peptides and proteins. Sixty-five unique peptide peaks were observed with changed expression level. Among them, nine distinct proteins, including CLCA2, GSTZ1, RUVBL2, NCOA7, MAAI, DNAJC14, NCKAP5L, CAPSD, and TEX2 were positively identified.

CLCA2, a target of the p53 family, negatively regulates cancer cell migration and invasion [21], and its knockdown causes epithelial-to-mesenchymal transition (EMT) [22]. GSTZ1, conserved over a long evolutionary period, plays a key role in the catabolism of phenylalanine and tyrosine [23]. In addition, GSTZ1 modulates sensitivity of cancer cells to dichloroacetate by controlling chloride concentrations [24]. RuvBl2 can cooperate with Ets2 to regulate the transcription of hTERT in colon cancer [25], and enhance tumor cell viability in HCC [26].

NCOA7, also known as ERAP140, was originally isolated on the basis of its interaction with ERα in an agonists-dependent manner. The non-canonical LXXLL motif in the central domain of NCOA7 is responsible for its interaction with ERα [27]. In addition to ERα, ERAP140 also shows specific ligand-inducible interactions with TRβ, PPARγ and RARα. Though it is reported that NCOA7 is recruited to the promoter region of ERα target gene following estradiol treatment in a dynamic fashion, NCOA7 has less sequence or structure homology with other co-activators, and might function as a distinct co-activator class [28]. In addition, NCOA7 contains a TLDc domain, which is highly similar to its homologue, oxidation resistance protein 1 (OXR1) [29]. Like OXR1, NCOA7 is capable to sense the oxidative stress and regulate cellular responses to oxidative DNA damage [30].

Relatively little is known regarding the involvement of NCOA7 in tumorigenesis. It is shown by genome-wide association studies that several gene polymorphisms of NCOA7 were associated with the development of breast cancer [31, 32]. Recently, individual studies demonstrated that NCOA7 regulated all-trans-retinoic acid (ATRA)-mediated neuronal differentiation and functioned as a favourable prognostic indicator for neuroblastoma [33]. In present data, MALDI-IMS revealed that NCOA7 was up-regulated in the OSCC tissue compared to OSF counterparts. The MALDI-IMS data was well validated by immunostaining and western blotting results. We showed that expression of NCOA7 was elevated in OSCC tissues, and was correlated with tumor location, tumor histological differentiation or lymph node metastasis. Further, we demonstrated that overexpression of NCOA7 induced OSCC cell proliferation in both cell and animal models. These data suggested that NCOA7 was a potential biomarker that could be used to detect OSF malignant transformation and further assist in the diagnostic and therapeutic decisions.

The AHR is a ligand-activated transcription factor that plays an essential role in the xenobiotic detoxification by regulating a series of drug-metabolizing enzymes. Accumulating studies showed that, AHR induced cell cycle arrest and decreased cell viability upon treatment with exogenous ligands. It was reported that 2,3,7,8-tetrachlorodibenzo-p-dioxin (TCDD), the best known high-affinity AHR ligand, retarded chicken coronary development through inhibiting myocyte proliferation [34]. Further, another AHR agonist 2-(4-amino-3-methylphenyl)-5-fluorobenzothiazole also induced DNA damage and cell cycle arrest in breast cancer cells in an AHR-dependent manner [35]. However, recent studies shed a light on a ligand-independent pro-proliferative property of AHR. It was observed that the number of proliferating fibroblasts from AHR-null mice embryo was substantially decreased compared to normal mice [36]. Moreover, siRNA-mediated AHR knockdown retarded cell cycle in HepG2 cells and resulted in a delay in the transition from G1 to S phase [37]. More recently, AHR was found to be upregulated and activated in OSCC compared to non-cancerous counterparts. Knockdown of AHR reduced tumor cell growth and tumorsphere formation [38]. In this study, we showed that NCOA7 bounded to either AHR or the promoter region of AHR's target gene, CCND1, in the nucleus of OSCC cell line. Notably, NCOA7 enhanced transcriptional activity of AHR and induced expression of CCND1 in absence of ligands, suggesting that NCOA7 was a ligand-independent coactivator of AHR. Further, we also demonstrated that loss of AHR deleted NCOA7 binding to CCND1 promoter and markedly abolished NCOA7-mediated OSCC cell proliferation. Therefore, it is reasonable to infer that NCOA7-induced OSCC cell proliferation is probably mediated by activating AHR and CCND1 in a ligand-independent way.

Efforts have been made to illustrate the mechanisms underlying development of OSCC from pre-malignant lesions, but few factors and pathways have been identified thus far. The current study suggests that NCOA7 is a potential diagnostic biomarker for OSCC arising from OSF, and NCOA7 promotes OSCC cell proliferation through potentiating AHR. Identifying this unrecognized oncogenic protein in OSCC might reveal a novel target for drug design and provide an opportunity for therapeutic intervention in oral cancer. Nevertheless, further study will be conducted for a more detailed validation of NCOA7 expression in OSCC, OSF and other OSCC premalignant lesions, by using a large scale of clinical samples. In addition, more work is still needed to decipher the precise mechanism that enables NCOA7 binding to AHR.

## MATERIALS AND METHODS

### Patients and tissue specimens

Thirty-four OSCC tissues arising from OSF and twenty-four OSF tissues without malignant transformation were obtained, respectively, from the Hospital of Stomatology, Sichuan University, with informed consent from patients. No preoperative treatment was performed in prior to sampling. Clinicopathologic stages of the OSCC patients were determined by two pathologists according to the TNM classification criteria defined by the American Joint Committee on Cancer (AJCC) [39]. Ten OSCC samples (including the adjacent OSF areas) were selected for MALDI-IMS analysis. These tissues were snap-frozen after surgery and stored at −150°C. Four OSCC tissues and four OSF tissues were collected for western blotting and stored in −80°C. Other forty specimens were fixed with 4% paraformaldehyde and embedded in paraffin for further immunohistochemical analysis. This study was approved by the Institutional Ethics Committee of Sichuan University.

### Antibodies

All the antibodies used in the study were purchased from Abcam: anti-NCOA7 (ab103993, 1/50), anti-Ki67 (ab15580, 1/100), anti-AHR (ab2770, 1/500), anti-CCND1 (ab6152, 1/500), and anti-beta Tublin (ab6046, 1/500).

### MALDI-IMS

MALDI-IMS was performed according to previous reports with minor modifications [40]. The principle workflow was summarized in Figure 1A.

#### Sample preparation

Frozen tissues were sliced into 10 μm sections using Leica CM 1900 cryostat (Leica Biosystems, Nussloch, Germany) at −20°C and thaw-mounted onto conductive indium-tin-oxide (ITO) coated glass slides (Bruker Daltonics, Bremen, Germany). The sister section was transferred onto a normal glass slide for hematoxylin and eosin (H&E) staining to check the adequacy of the frozen material. Mounted tissue slices were air-dried in a desiccator for 1h. ITO slides were then washed with ethanol solutions (twice with70% ethanol for 1 min each and then once with 95% ethanol for 30 s) and then were dried again in a desiccator for 30 min. A MALDI matrix solution (30 mg/ml DHB in acetonitrile/0.2% trifluoroacetic acid) was sprayed uniformly over the section using the ImagePrep matrix application device (Bruker Daltonics) following the standard protocol, which controls matrix deposition and thickness of the matrix layer.

#### MALDI imaging

Digital images of the matrix-sprayed tissue slides were collected with a flatbed scanner prior to MALDI analysis. The sister normal slides were stained with H&E and scanned with an automated slide scanner (Scanscope, Aperio, USA). The areas for MALDI Imaging were selected on matrix-sprayed slides based on H&E staining image of their corresponding sister slides. The matrix-sprayed slides were then introduced into the Ultraflex TOF/TOF mass spectrometer (Bruker Daltonics) and mass spectra were acquired in the mass range m/z 800-10,000 at the sampling rate of 0.13 GS/s across the entire tissue area in positive linear and reflectron ion mode. Approximately 500-1000 laser shots were accumulated and averaged per laser spot. A laser spot diameter of 50 μm and a raster size of 100 μm were employed. Acquisition and visualization was carried out by FlexImaging software, version 2.0 (Bruker Daltonics) in order to spatially relate the peak intensities to histological structures.

#### Data acquisition and analyses

Extracted mass spectra underwent calibration on common “background” peaks (spectral alignment) and normalization with root mean square, vector norm algorithm, which provided a very uniform distribution of intense signals based on their total ion count (TIC) in the observation mass range utilizing ClinProTools 3.0 software (Bruker Daltonik). The OSCC and OSF areas for comparing the peak intensity were selected based on the H&E staining image, respectively. Wilcoxon rank-sum test was applied to evaluate the significant differences in peak intensities between OSCC and OSF groups with a significance cutoff of *P* <0.05. The resulting significance values were corrected by Benjamini-Hochberg for multiple testing on a single data set and principle component analysis (PCA). PCA is one of the most used approaches to extract the interesting features from a data set, which is established as a statistical method commonly used to reduce the dimensionality of a multivariate data set. Visual inspection identified histologically heterogeneities between the histological groups and the best discriminatory m/z peaks in the mass rang m/z 800-1,500 were selected for further analyses.

After ClinProTools analyses, the highly expressed area for an individual peak was selected on the corresponding matrix-sprayed sister slide, and was used for further MS/MS identification. The selected peaks were sent to MS/MS list and the mass spectrum was manually transformed to the LIFT model. Each of these peaks was solely set as the parent ion and 10000 laser shots were accumulated after transition to the fragments model. The MS/MS results were confirmed by BioTools and MASCOT (Matrix Science). The MASCOT search was set as: the taxonomy was Homo sapiens; the significance threshold was set to 0.05; the MS/MS error tolerance was 0.5 Da; no enzyme cleavage. For each protein, a max score, a rank, an E-value, a calculated m/z value and mass number were given. The genetic information of proteins with highest score was processed for bioinformatics analysis in the context of molecular networks and to explore the complex physiological role of the proteins.

### Bioinformatics analysis

#### Network construction

To build the protein-protein interaction (PPI) network based on the significantly differentially expressed proteins finding in the MALDI-MS, we collected biological evidence from PrePPI [41]. After that, network related proteins were assigned to 10 functional synaptic protein groups by Gene Ontology (GO) Annotation clustering, which was performed using DAVID database (http://david.abcc.ncifcrf.gov/) [42, 43]. Enrichment was only considered relevant when over represented functional groups contained at least 100 proteins. The cell cycle and cell proliferation subnetworks were assigned and the unified conceptual framework of PPI network was integrated by Cytoscape [44].

#### Function clustering and pathway analysis

Functional enrichment was determined using the DAVID functional annotation tool. GO term related to Biological Process (BP), as well as pathway annotations derived from Kyoto encyclopedia of genes and genomes (KEGG) were used in the functional categories [45–47]. All differentially expressed proteins being in the form of UniProt accession numbers, the functional annotation analysis was performed with the gene ontology tool (GOTERMCCALL). Only those GO terms yielding a P < 0.05 using a Fisher's exact test were considered significantly enriched in each gene list while pathways with a corrected P < 0.05 were considered significant, classified into hierarchical categories according to KEGG.

### Immunohistochemistry

Immunohistochemical staining was performed following previous reports [48]. The slides were stained using the Envision System horseradish peroxidase method (DakoCytomation Inc, Carpinteria, CA) according to the manufacturer's instructions. Sections were deparaffinized in xylene and hydrated in a descending series of ethanol concentrations and endogenous peroxidase activity was quenched by incubation with 3% H_2_O_2_ for 5 min at room temperature. Antigens were retrieved by boiling the sections in 0.01 M Ethylene Diamine Tetraacetic Acid buffer (EDTA), three times for 5 min in a microwave at 350 W. Sections were permeabilized and nonspecific epitopes were blocked by incubation for 30 min in PBS containing 5% BSA. Sections were probed overnight with anti-NCOA7 and followed with anti-rabbit biotinylated secondary antibody for 1 h at 37°C. Finally, sections were visualized with 2, 3-diaminobenzidine (DAB) hydrochloride.

To score the slides, at least eight individual fields in one slide were chosen, and 100 cells were counted for each field. Cells with nuclear NCOA7 immunoreactivity were considered as positive cells. The score for each slide was measured as the cross product of the value of immunostaining intensity and the value of proportion of staining-positive cells, as described previously. Immunostaining intensity was divided into five grades: 0, negative; 1, weak; 2, moderate; 3, strong; 4, very strong. The proportion of staining-positive cells was divided into five grades: 0, <5%; 1, 6 –25%; 2, 26 –50%; 3, 51–75%; 4, >75%. The results (the grade of immunostaining intensity multiplied by the grade of the proportion of staining-positive cells) were defined as: 0-4, low; 5-16, high. Results were assessed and confirmed by two independent experienced pathologists.

### Cell culture

Five OSCC cell lines, HSC-3, HSC-4, Cal-27, Cal-33 and SCC25 were used in this research. HSC-3 and HSC-4 were purchased from the cell bank of Japanese Collection of Research Bioresource (JCRB, Shinjuku, Japan). Cal-27 and SCC25 were purchased from American Type Culture Collection (ATCC, Manassas, USA). Cal-33 was obtained from the European Collection of Cell Cultures (ECACC). HSC-3 and HSC-4 cells were cultured in DMEM (Gibco, Eggenstein, Germany) supplemented with 10% fetal bovine serum (FBS; Gibco), and Cal-27, Cal-33 and SCC25 cells were maintained in DMEM/Ham's F-12 (1:1) supplemented with 10% FBS and hydrocortisone (400 ng/ml) (Sigma-Aldrich, Poole, UK) under 5% CO2 and humidified air atmosphere at 37°C.

### siRNA interference

Transfection was performed with Lipofectamine 2000 reagent (Invitrogen, Rockville, MD, USA) following the manufacturer's protocol. SiNCOA7 was purchased from Dharmacon, and other siRNAs were synthesized according to previous reports [49–51].

### Cell proliferation assays

For BrdU labeling assay, cells were seed in 96-well plate. After treatment, cells were incubated with 10 mM BrdU (Roche) for 12 h at 37°C and then the labeling medium was carefully removed. The cells were fixed with 200 μl pre-cooled fixative per well for 30 min at −20°C. After removing fixative, anti-BrdU-POD working solution was added to each well and kept at room temperature on a shaker for 60 min. BrdU signal was measured by using 5-Bromo-2′-deoxy-uridine Labeling and Detection Kit III (Roche).

For colony formation assay, after treatment, 100 cells were seed in six-well plate. After incubation for 14 days at 37°C in a humidified chamber containing 5% CO_2_, the cells were washed with normal saline and fixed in 75% ethanol. Then the colonies were counted after being stained with crystal blue for approximately 30 min. Those clones containing more than 50 cells were defined as positive clones.

### Nucleus isolation

Cells were sedimented at 800 g for 10 min and then washed with 50 vol of PBS. Then the pellet was resuspended in a 5x packed cells volume of a hypotonic buffer (10 mM HEPES, pH 7.9, 0.75 mM spermidine, 0.15 mM spermine, 0.1 mM EDTA and 0.1 mM EGTA). After the supernatant was removed and replaced with 2 vol of fresh hypotonic buffer plus PI cocktail, cells were homogenized by 3-5 strokes in a homogenizer and sucrose restoration buffer was added (1 vol of 500 mM HEPES, pH 7.9, 7.5 mM spermidine, 1.5 mM spermine, 2 mM EDTA, 2 mM EGTA and 10 mM DTT in 9 vol of 7.5% sucrose). Nuclei were sedimented in a centrifuge for 30 s at 14 000 rpm.

### Western blot

Protein lysates from the indicated cells were collected in RIPA buffer (50 mM Trisbase, 1.0 mM EDTA, 150 mM NaCl, 0.1% SDS, 1% TritonX-100, 1% sodium deoxycholate, 1 mM phenylmethylsulfonyl fluoride) and quantified with the DC Protein Assay Kit (Bio-Rad Laboratories). After the total protein was determined, an aliquot of 10 μg of proteins was subjected to 10% or 12% SDS-PAGE under reducing condition and the gels were then transferred to PDVF membranes. After incubation with 5% skimmed milk at room temperature for 1h, the membranes were further incubated overnight with primary antibodies at 4°C. After washing with PBST, the membranes were incubated with horseradish peroxidase-conjugated secondary antibodies. Immunoblot signals were visualized using ECL Western blotting detection reagent (Millipore).

### Chromatin immunoprecipitation

Chromatin immunoprecipitation (ChIP) assays were performed using the ChIP-IT Express Enzymatic kit (Active Motif, Carlsbad, CA, USA) based on the protocol provided by the supplier. Briefly, cells were fixed with 37% formaldehyde solution for 10 min at room temperature, and the reactions terminated by the addition of glycine. The cells were lysed to release the nuclei and centrifuged at 2400g at 4°C for 10 min to pellet the nuclei. Chromatin was resuspended, sonicated into 300-400 bp fragments, and then probed with antibodies. The immuno-adsorbed DNA was purified by phenol/chloroform extraction followed by ethanol precipitation, and was then analyzed by PCR using the following primers: CCND1, forward, 5′-GCT CCC ATT CTC TGC CGG-3′; reverse, 5′-CGG AGC GTG CGG ACT CTG-3′ [17].

### RT-PCR

Total RNA was extracted with a single step method using the TRIzol reagent (Life Technology). Primer synthesis and RT-PCR was performed by following previous reports [52].

### Statistics

Statistical analysis was performed using SPSS 19.0 (SPSS, Chicago, IL). The quantitative variables were calculated by one-way ANOVA or Student's t-test when appropriate and categorical variables were calculated by Wilcoxon test. For all tests, *P* < 0.05 was defined as the level of significance.

## SUPPLEMENTARY FIGURE


