# Supplementary material for: MALDI imaging reveals NCOA7 as a potential biomarker in oral squamous cell carcinoma arising from oral submucous fibrosis
Source: Oncotarget. 2016 Aug 4;7(37):59987–60004. doi: 10.18632/oncotarget.11046 (PMC5312364; doi:10.18632/oncotarget.11046)
Supplement: Supplementary file 1 [file oncotarget-07-59987-s001.pdf]

MALDI imaging reveals NCOA7 as a potential biomarker in oral squamous cell carcinoma arising from oral submucous fibrosis

SUPPLEMENTARY FIGURE

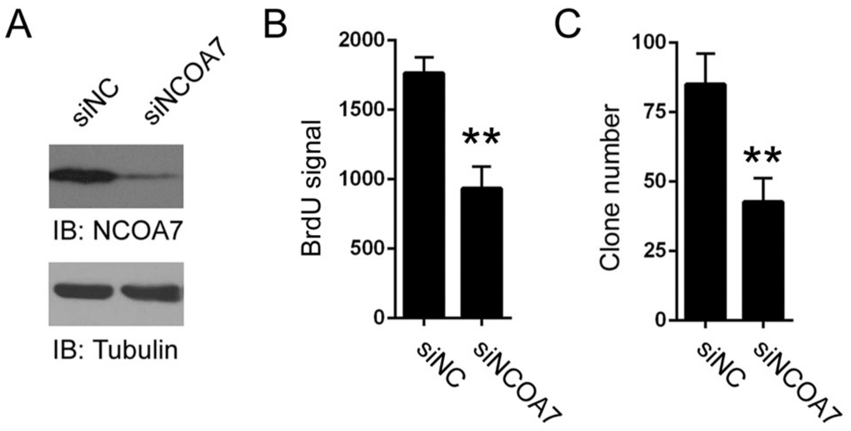

Supplementary Figure S1: A. HSC-4 cells were treated with siNC or siNCOA7, and expression of NCOA7 was examined by western blot. **B.** HSC-4 cells were treated with siNC or siNCOA7, and proliferation HSC-4 cells were examined by BrdU labeling assay. **C.** HSC-4 cells were treated with siNC or siNCOA7, and proliferation HSC-4 cells were examined by colony formation assay. All data were representative of at least three independent experiments. **, $P < 0.01$.
